# “You better use the safer one… leave this one”: the role of health providers in women’s pursuit of their preferred family planning methods

**DOI:** 10.1186/s12905-020-01034-1

**Published:** 2020-08-12

**Authors:** Robel Yirgu, Shannon N. Wood, Celia Karp, Amy Tsui, Caroline Moreau

**Affiliations:** 1 Department of Reproductive Health and Health Service Management, School of Public Health, Addis Ababa University, Addis Ababa, Ethiopia; 2 Department of Population, Family and Reproductive Health, Johns Hopkins Bloomberg School of Public Health, Baltimore, USA; 3 Soins et Santé primaire, CESP Centre for Research in Epidemiology and Population Health U1018, Inserm, F-94805 Villejuif, France

**Keywords:** Contraception, Preferences, Empowerment, Health provider, Provider bias

## Abstract

**Background:**

Universal access to quality sexual and reproductive health (SRH) services is pivotal to ensuring gender equality. In high-income countries, patient-provider interactions have been shown to shape women’s decisions about contraception, with poor exchanges decreasing method uptake and satisfaction. While significant progress has been made to increase women’s access to SRH services, in low- and middle-income countries, little is known about the quality of family planning patient-provider interactions. The primary objective of this analysis was to explore the role of health care providers in women’s family planning decision-making in Ethiopia.

**Methods:**

From July to August 2017, 10 focus group discussions (*n* = 80) and 30 in-depth interviews were conducted with women aged 15–49 and men aged 18+ recruited via purposive sampling from urban and rural sites in Ethiopia. Semi-structured interview guides explored women’s and girls’ empowerment in SRH surrounding sex, childbearing, and contraception. All interviews were conducted in Amharic, audio-recorded, and transcribed verbatim into English. Inductive thematic analysis was used to analyze data. Eleven codes specific to provider services for family planning were reviewed and matrixes creates for synthesis.

**Results:**

Three primary themes emerged: the role of providers in women’s awareness of and demand for family planning services; selection and uptake of contraceptive methods; and discontinuation and switching of contraceptive methods. Results indicate that health extension workers were central to women’s awareness of family planning, and health providers’ endorsements were instrumental in decisions to adopt methods. The majority of respondents described positive interactions with providers and appreciated thorough counseling when considering using or switching methods. Some women, however, described health providers directing them toward long-acting methods by communicating inaccurate information or emphasizing disadvantages of short-acting methods. A few women described provider reluctance or resistance to switching methods, especially from implants.

**Conclusions:**

Women shared many narratives about the central roles health providers played in their awareness and decision-making for family planning. Those narratives also included provider bias against women’s preferred methods. Further research and program assessments are needed to ascertain the extent to which these biases hinder women’s decision-making autonomy in using contraception.

## Background

Gender equality is a human rights imperative and a pillar of development, as outlined in Sustainable Development Goal 5 (SDG-5) [1]. To achieve gender equality, universal access to quality sexual and reproductive health (SRH) services is essential [2]. Globally, however, only half (57%) of married women aged 15–49 are free to make decisions about sexual relations, contraception, and SRH services [3]. Increasing women’s autonomy in SRH decision-making not only increases access to SRH services, but may also have a cascading, and potentially bidirectional, influence, in decreasing adverse maternal and child health outcomes and increasing women’s involvement in social, economic, and political spheres [4–6].

High-quality services and provider-patient interactions are central to women’s SRH decision-making [7, 8]. Providers play an important role in ensuring that women obtain accurate information, have access to a range of contraceptive methods, and make informed decisions when selecting their contraceptive method of choice—or no method at all [9, 10]. However, the quality of healthcare interactions varies by provider, leading to disparities in counseling and provision of services for family planning [9, 11, 12]. Poor provider interactions may result in decreased use of health services, and higher discontinuation and dissatisfaction with services [13–15]. Understanding the ways that women’s interactions with health providers shape their family planning decision-making is important to maximizing the quality and use of family planning services for all women throughout the life course.

One key principle of high-quality client-provider interactions related to family planning services is provider respect for women’s fertility plans and family planning preferences [16]. Global and country-specific targets calling for increased contraceptive use to reduce unmet need for family planning, including use of longer-acting methods, have posed challenges to ensuring quality counseling on all family planning methods and respect for women’s use of preferred methods, while aiming to achieve these targets [17–19]. As a response, recent research and practice has renewed attention towards improving the quality of reproductive health care services through emphasis of client-centered care [8, 19, 20]. To date, however, there is a dearth of research on quality of family planning services specific to low-and middle-income countries (LMICs), where achieving national demographic targets may take precedence over women’s individual fertility intentions and contraceptive preferences [19, 21–23].

Ethiopia is a focus country of Family Planning 2020 (FP2020), with a commitment to increase the national modern contraceptive prevalence rate (mCPR) to 55%, decrease total fertility rate (TFR) to 3 children per woman, and add 6.2 million additional family planning users by 2020 [24]. While these goals are ambitious, Ethiopia achieved significant progress between 2014 and 2018, with a four-percentage point increase in mCPR from 33.8 to 37.8% [25]. This breakthrough is in part due to improved access to family planning services through the expansion of primary health care to Ethiopia’s rural population [24]. Further, this expansion coincided with the adoption of task-shifting policies that aimed to increase coverage of family planning services by shifting service provision from clinicians to trained mid-level health care providers and Health Extension Workers (HEWs) [24, 26].

The rapid development of Ethiopia’s health system and the expansion of family planning services offer a research opportunity to understand the quality of client-provider interactions and assess how women negotiate to meet their contraceptive goals during these interactions. This study is a secondary analysis of data collected for a multi-country study, which aimed to investigate women’s and girls' SRH empowerment in three sub-Saharan African countries. The present analysis explores the role of providers in women’s family planning decision-making in Ethiopia.

## Methods

### Overview

The Women's and Girls' Empowerment for Sexual and Reproductive Health (WGE-SRH) study was conducted to develop a cross-cultural quantitative index to assess dimensions of female empowerment over sexual activity, contraception, and pregnancy domains. Qualitative data collection occurred under a multi-site collaboration from July to August 2017 in four sites in three countries: Ethiopia, northern and southern Nigeria, and Uganda. The current study focuses on Ethiopia only and was implemented by researchers from the Addis Ababa University (AAU) School of Public Health in partnership with researchers from the Johns Hopkins Bloomberg School of Public Health (JHSPH). Ethical clearance was obtained from the Institutional Review Boards (IRBs) at JHSPH and AAU.

### Study setting

The study was conducted in the North Shewa Zone, approximately 130 km north of Addis Ababa. Rural and peri-urban areas were selected within this zone primarily for convenience purposes given proximity to Addis Ababa.

To understand the role of the health providers in enabling women’s use of their preferred family planning methods, an understanding of the government and health system structures is helpful. The health system is organized into a three-tier system (i.e. primary, secondary, and tertiary) [27]. The tertiary level comprises specialized and teaching hospitals, whereas the secondary level consists of general hospitals. The primary level includes a primary hospital, one health centre, and five health posts. Health posts are the health system entities at the grassroot level, where each *kebele* (the lowest administrative unit comprising 500 households) is expected to have one health post. These facilities are staffed with two HEWs, community health workers and the frontline actors in the primary health care expansion program. They are trained for one year on disease prevention and health promotion surrounding 18 primary health care packages, including family planning services [26]. Family planning counseling and provision, including the insertion and removal of implants, are services generally rendered by HEWs, with higher level services requiring referral or consultation to health centres [28]. The primary health system further has an auxiliary community-based extension called the 1-to-5 structure; this structure aims to enhance health literacy through regular discussions among female community group members, on selected health topics [27].

### Training

Data collectors included eight Ethiopian men and women with advanced degrees in public health, previous experience in qualitative research, and fluency in Amharic. All data collectors completed a five-day training co-led by JHSPH and AAU staff in July 2017. Training topics included qualitative interviewing, research objectives, interview guides, ethics, and transcription/translation. Mock sessions and pilot testing of the interview guides, adapting them to the Amharic language and Ethiopian social and cultural norms while maintaining content consistency across sites, was implemented as part of the training. All data collectors, principal investigators, and study staff completed human subjects training. Following pilot testing, four data collectors, who also served as coders, completed an additional three-day training to discuss preliminary themes, develop a codebook structure, and enhance their Atlas.ti coding skills.

### Recruitment

Participants were recruited via community-based sampling, with the assistance of local gatekeepers. Once households were identified, gatekeepers within the communities provided initial information to participants to introduce women to the study objectives and sensitive interview topics. Potential participants were then given the opportunity to contact the in-country principal investigators with any questions or concerns about the study or their participation. Once interest was expressed, the trained interviewers conducted eligibility screening and verbally consented each woman privately; consent was obtained from all participants. Consenting women were asked for permission before the team approached their partners. Oral consent followed in-country IRB guidelines; head of household consent and child assent was obtained for females age 15–17. Eligibility criteria comprised female residents of the study area aged 15–49 and men whose wives were aged 15–49. Purposive sampling by age, sex, and geographic area (urban/rural) was utilized to ensure maximum variability.

### Procedures

Thirty in-depth interviews (IDIs) and 10 focus group discussions (FGDs) were conducted with women aged 15–49 and men age 18+. FGD sampling did not necessitate that men and women were in partnership. For IDIs, 12 couples were included (12 men and 12 women) and interviewed separately, as well as an additional six unmarried women across age groups and residences. Both FGDs and IDIs used semi-structured interview guides. The guides, developed for the WGE-SRH parent study, covered topics that aimed to capture women’s and girls’ empowerment in sexual and reproductive health constructs, including autonomy, self-efficacy, and agency, related to sex, childbearing, and contraception (Supplementary File 1) [29]. FGD guides focused specifically on community perspectives of these topics, whereas IDI guides focused on the personal experiences, perspectives, and narratives of women, girls, and their male partners.

FGDs were conducted in private settings at community centres or designated facilities; IDIs took place in private settings at the local partner’s offices or at convenient locations for the participant. To ensure ease of discussing potentially sensitive topics, moderators were gender-matched to participants. Each FGD and IDI took approximately 60 to 90 min. After completion of FGDs/IDI, universal upset screeners were administered to all participants individually to ensure that questions did not illicit emotional distress [29, 30]. If distress was observed, participants were referred to local health facilities for psychosocial support. Both the FGD and IDI participants were given 100 Ethiopian Birr (approximately US$3.5) for their time participating in the study.

FGDs with 10 individuals per group were conducted for women by age group and separately for adult men aged 18 and older. FGDs were further stratified by urban/rural residence. Male and female FGDs were not linked and eligibility criteria did not necessitate partner inclusion. Each FGD had a moderator and notetaker.

IDIs were conducted with individual partners from twelve couples (men and women), as well as six additional single women per site (*n* = 30 per site for a total of *n* = 120 IDIs across four sites). Couples were stratified by urban or rural residence and female age group to allow for a diverse representation; six additional unmarried women were interviewed to explore empowerment outside of marriage. Given the potentially sensitive nature of interviewing couple dyads, IDI consent was first obtained from the female partner. After her interview, she then decided if she wanted her husband to be approached for interview, though no participants declined partner’s participation.

### Analysis

Interviews were conducted in Amharic and audio-recorded. The interviewers transcribed their respective interviews, verbatim, into English. An inductive thematic approach was used to analyze the data, including familiarizing oneself with the data, generating initial codes, sorting the data, and reviewing themes and sub-themes identified in the analysis. This analytic approach was selected in order for themes to emerge directly from the quotes of participants [29, 31]. A team of investigators from JHSPH led the cross-site codebook development, which involved a rigorous review of the transcripts and frequent discussions with in-country teams in Ethiopia, Nigeria, and Uganda. Four Ethiopian coders, well-versed in qualitative research and trained by the JHSPH team, coded the transcripts using Atlas.ti software, employing the codes outlined in the cross-site codebook. For themes distinctive to Ethiopia, country-specific codes were developed and analyzed separately.

One hundred sixty codes were used to analyze the data from the broader WGE-SRH study, which sought to understand women’s empowerment in SRH decisions. For this analysis, we selected 11 codes specific to women’s interactions with healthcare providers who deliver family planning services in Ethiopia (Table 1). All quotes identified by the selected codes were reviewed and organized into categories. The data from these categories were analyzed to develop the emergent sub-themes. Sub-themes were then grouped into three overarching themes based on regular investigative team discussions.

**Table 1:** Codes used for the present analysis

familyplan_benefits	familyplan_method_womanchoice
familyplan_barriers	familyplan_method_provider
familyplan_misconception	familyplan_method_discussed_provider
familyplan_info_healthprovider	familyplan_experience_positive
familyplan_comfortaccess	familyplan_experience_negative
familyplan_method_comfortdiscuss_provider	familyplan_method_nonegotiate_provider
familyplan_benefits	familyplan_method_notdiscussed_provider
familyplan_method_negotiate_provider	

## Results

Results are structured around three emergent themes: 1) role of providers in women’s awareness of and demand for family planning services; 2) role of providers in women’s selection and uptake of family planning methods; and 3) role of providers in women’s discontinuation and switching of family planning methods.

### Role of providers in women’s awareness of and demand for family planning services

#### Health workers regarded as knowledgeable and trusted sources of family planning information

Most respondents regarded providers as benevolent healthcare actors who were responsible for maintaining the health of people in the study community. One female respondent noted:

*From the beginning, health care providers are here to help people. I think they will teach things that are good for people and I don’t think they bring any bad thing to people.*

--*Female IDI participant, 26 years age, Rural*

Trust in providers was communicated as central to protecting health. One respondent noted that failing to discuss contraception, and broader health matters, with the providers would be dangerous for their well-being: *It is good to discuss with health professionals about everything. Without them, there will be a lot of damage (Female IDI participant, 29 years age, Rural).*

Although women noted different sources to obtain information about family planning, healthcare providers were perceived as the most credible. Many female participants described their own processes of learning about and considering whether or not to use contraceptives. Women reported sharing their initial thoughts about general contraceptive use and use of specific methods with their partners, friends or parents; however, the information they obtained from these sources was only considered accurate after they consulted with their providers:

*My grandmother doesn’t know anything, so I think it is good or more appropriate if I discuss with the HEW because she knows what is good or bad for me.*

*--Female FGD participant, 25-29 years age, Rural*

#### HEWs were primary family planning educators and promoters

Many participants identified HEWs, mid-level health providers, and the 1-to-5 platform as primary educators and promoters of family planning methods. Respondents noted that HEWs were instrumental in helping to generate awareness by leading house visits, educational campaigns, and individual counseling to educate women about the range of family planning methods available; fewer discussions surrounded the contribution of mid-level health providers and the 1-to-5 structure.

*In this community around 95% of women use family planning. This happened after HEWs started to work in our kebele. Previously people chose not to use contraceptives on the basis of their religious beliefs. But these days, only few disagree with HEWs concerning the use of contraceptives. Some women even ask apologies from the HEWs for their previous resistance.*

*-- Male FGD participant, 40 years age, Rural*

### Role of providers in women’s selection and uptake of family planning methods

#### Many women were comfortable receiving family planning services from providers

Women shared both positive and negative experiences of interacting with family planning providers. In general, most women were satisfied with their encounters with health care providers. The detailed information that health care providers shared with women about the different family planning methods during counseling sessions was one of the reasons for their satisfaction. For example, as one respondent noted:

*They will tell you if [the family planning method] has any side effects and also the benefit. They will also tell you for how long it works. I think it is beneficial to go to the hospital or discuss with HEWs in the kebele.*

*--Female IDI participant, 15 years age, Urban*

Women also reported positive client-provider interactions because the providers respected their fertility intentions, which in turn informed their use of family planning methods.

*Discussing family planning with providers is good. For example, if I go to a health facility and tell them that I want to wait for a while before giving birth, they won’t oppose my decision…they will say "ok".*

--*Female IDI participant, 20 years age, Rural*

Some women indicated disagreement with their partners on fertility decisions. Despite these discordant fertility intentions, women highlighted the ways in which providers supported them in controlling their own fertility. In most situations where women disagreed with their partners, women were comfortable discussing their circumstances and contraceptive options with providers. Respondents described their confidence that providers would recognize their own reproductive autonomy despite their partners’ conflicting opinions: *If he refuses, I will take my own measure. Since I don’t have to be burdened with children, I will discuss and agree with the health [HEW]* (*Female IDI participant, 29 years age, Rural).*

#### Some women feared or distrusted healthcare providers about family planning

While the majority of the participants claimed they were comfortable with the services and interactions with health care providers, some participants reported distress or discomfort in interacting with providers about family planning. For some women, this negative feeling originated in a lack of orientation about the facility and the procedure:

*I was frightened to even talk with the health workers...she asked why I was shy. Then they took me to the other room, and I got more scared because I thought the procedure would hurt.*

*--Female IDI participant, 25 years age, Urban*

Women also reported apprehension when their own opinions about family planning differed from their providers. Some feared that their opposing views could negatively affect their relationship with the providers and the care they received. As a result, women reported negotiating to protect their own preferences regarding side effects, desired length of use, and fit to their specific circumstances, while trying not to offend the providers.

*I would be “seme ena work” [an Amharic expression for an absolute agreement] with the providers. We don’t like to use pills, but the providers tell us to use pills. But I tell them that pills don’t work for me, I will try to work with them, but if I refuse to change my mind [in the selection of* a method*], the provider will be rigid too. Instead, I will explain my condition, explaining that my husband will throw away the pills if he finds it, this way, she will sympathize with me. But if I insist on getting another method, ignoring her recommendations, we cannot agree and she will send me home with no family planning method.*

--*Female IDI participant, 29 years age, Rural*

Further, while reported less frequently, some women also described health providers asking whether the woman’s partner agreed to her use of contraception before being willing to provide the method.

*When she goes to the hospital, the first thing they will ask her is if she is sure about using family planning, then they will ask her if she has discussed with her husband.*

*--Female IDI participant, 26 years age, Rural*

#### Some women felt manipulated towards using long-acting reversible contraceptives (LARCs)

Although some women described resolving their discordant opinions about family planning methods with providers, other women reported that providers insisted on the use of particular methods regardless of their method preference. Additionally, women sometimes felt that providers overlooked their preferences in favor of other methods, particularly a longer-acting method like the implant:

*The HEW insisted that I use the five years implant instead of taking the injectable every three months. But I refused to start using the implant and left the health post.*

--*Female IDI participant, 18 years age, Rural*

Women reported manipulation, often through inaccurate information about the characteristics and side effects of short-acting methods, wherein providers could shape women’s understanding of the full range of family planning methods in favor of specific methods (e.g., implants). As one female respondent noted:

*The HEW told me that the injectable causes narrowing of the uterus and if I want to give birth later, I will need an operation [caesarean section]. [The HEW said]: “You want this? (laugh)…You better use the safer one. Leave this one, use the three years; the five year might be a bit long. Just leave the three-month [method], it harms you.”*

--*Female IDI participant, 18 years age, Rural*

In other instances, respondents noted that providers emphasized disadvantages of short-acting methods in order to guide clients towards using LARCs:

*When I asked for their advice, they often recommend the one inserted in the arm [the implant] and uterus [the IUD]. They say this one is [the injectable] is not reliable.*

--*Female FGD participant, 26 years age, Urban*

While many women reported difficulty resisting providers’ influence in their decision-making, some women were able to find means, such as negotiating use of the provider’s recommended method in the future, in order to use their preferred method at the time of the interaction:

*When the providers suggest that “if you want to have more space between your pregnancies you have to use this method”, but I don’t want to use the method he suggested, so I will ask him to let me use the method I want now, and tell him that I will use what he has proposed some other time.*

*--Female IDI participant, 29 years age, Rural*

However, most women accepted the recommended methods by the providers, despite the method being different from the method they wanted to use when they first came to the facility, and it was not always clear from these discussions whether women were satisfied with their method selection.

*Since we did not have enough money to start a family, my husband and I wanted to switch from pills to the injectable. So, we asked the provider for the injectable, but she told me that the injectable was not a good contraceptive. She suggested the implant, so we eventually agreed to use the implant.*

*--Female IDI participant, 27 years age, Urban*

Alternatively, some women sought family planning services from private facilities to obtain their preferred family planning method, if they felt they were not able to receive it at a public facility:

*I refused when they offered me the one that is inserted in the arm. I refused two or three times and it is difficult when they don’t agree with your interest. So now I am using the method from a private facility, paying for the service.*

*--Female IDI participant, 26 years age, Rural*

### Role of providers in women’s discontinuation and switching of family planning methods

#### Women were comfortable discussing their decision to discontinue or switch methods with their providers

A number of participants reported discussing their intentions to discontinue or switch methods with their provider before taking action. Women reported HEWs as allies in navigating these decisions, as they were regarded as a reliable source of information when women had doubts about specific family planning methods:

*[HEWs] counsel women on whether they have to continue using contraceptives or stop using contraceptives and give birth. Then the women will go home, discuss with their husbands and decide.*

*--Female IDI participant, 48 years age, Rural*

Side effects of family planning methods were the most common reasons for seeking providers’ opinions on discontinuing or switching the method they were using. Many participants described that providers would have solutions for managing side effects. As one female respondent noted:

*I may use the injectable and my period may disappear, or I may have other complaints using the implant, in that case I will discuss with the health care provider and ask why this is happening. I will consult her about the challenge I have faced, and she will give me a solution and if there is a serious problem, she will tell me to go to the hospital.*

*--Female IDI participant, 25 years age, Urban*

### Some women had difficulty switching between methods or stopping them completely

Despite women’s willingness to seek providers’ opinions and assistance related to method discontinuation or switching, providers’ responses to such requests were not always positive. Some women reported that providers resisted their requests to switch between methods, and at times, openly refused to allow women to change methods. In particular, women discussed challenges with implant removals, which required care from a skilled provider, as preventing them from discontinuing the method or switching to another method. As one respondent noted:

*What I know about the method [implant] is that they won’t remove it when its duration is overdue. They tell you to go to private facilities and have it removed, or they will give you some other reason.*

--*Female FGD participant, 26 years age, Urban*

More frequently, however, women illustrated healthcare interactions in which providers minimized their complaints and omitted the opportunity of switching methods from the client-provider discussions. For example, as one female respondent described her healthcare interaction:

*Ever since I began using the three years implant, I started to miss my period. That gave me a lot of negative thoughts, and I was worried. So, I went to the health center once or twice, and they told me it’s the implant that was causing this. They told me that sometimes it causes heavy bleeding and sometimes it just disappears.*

*--Female IDI participant, 25 years age, Urban*

## Discussion

These results highlight the critical role that providers play in women’s awareness, selection, and use of contraception in Ethiopia. While women may discuss family planning with a variety of sources, including partners, friends and family members, they widely reported preferring a trusted health professional’s endorsement prior to using contraception. Health providers were central to supplying women with information about specific types of methods and differentiating between methods that women may have heard about within their social networks. Further, results indicate that HEWs were viewed as the most approachable and trustworthy providers for initial contact in learning about family planning; similar avenues of family planning knowledge dissemination have been observed in other Ethiopian studies [32, 33], emphasizing the continued importance of expanding HEW services.

Many respondents described positive interactions with providers, however, several experiences were characterized as negative and warrant further exploration. Notably, some of the study’s findings related to healthcare providers’ actions to undermine women’s use of their preferred methods, for example, communicating inaccurate information about short-acting methods or emphasizing disadvantages of short-acting methods favoring long-acting methods. These results are mirrored in findings from other LMICs that discuss biased and directive family planning counseling [19, 23]. Further, providers’ resistance and refusal to accommodate women’s desires to switch methods violates women’s autonomy. This discussion must be understood within the broader context of health services, as providers may face challenges operating within and managing external constraints on their delivery of reproductive health services. Such influences may also shape providers’ attitudes and approaches to service delivery. Future research should explore provider motivations, including systemic pressures within facilities and by supervisors, that may limit women’s reproductive choices.

Women described providers as a safe outlet for voicing their own relationship and childbearing concerns and also as a helpful resource for navigating discordant family planning views with their partners. Some women, however, also indicated that providers asked for confirmation of partner knowledge of family planning use before providing them with their desired methods. Several women expressed discomfort from being questioned about partner approval or knowledge of use prior to receiving contraception which made them averse to receiving services from the providers involved. In light of recent findings on Ethiopian women’s use of contraception without partner knowledge and estimates that nearly one-quarter of women use covertly in Ethiopia [34, 35], the role of providers as mediators in couples' contraceptive decision-making should be further explored.

This study was not without limitations. Foremost, discussions surrounding provider involvement were intertwined with conversations on a number of other family planning actors, including family members and partners; as such, disentangling the specific provider under discussion (e.g. HEWs, nurse, midwife, physician) was sometimes challenging throughout interviews. Follow-up questions and mapping such discussions to specific cadres of providers in future research would allow for a more nuanced analysis by provider and method type. Further, community-based convenience sampling may have allowed for women who had more extreme experiences with family planning to self-select into the study; however, given that this study was broadly based on women’s pregnancy, family planning, and sexual experiences, this is unlikely. Generalizability of results is limited to women within urban and rural areas relatively close to the capital city of Ethiopia and should not be extrapolated to Ethiopia as a whole or other settings. Specifically, these findings may not apply to Ethiopia’s pastoral communities given unique cultural factors that affect women’s decision-making in those settings. Finally, the interview guides were developed to address the research questions of the parent study, not this specific investigation into client-provider interactions. The data from which this secondary analysis was conducted are limited to the discussions generated from questions in the parent study’s interview guides.

Nevertheless, these results are important and can inform the management and implementation of reproductive health programs to ensure that family planning practices are concordant with the services that women desire. First, our findings indicate that the majority of women not only want to access a broad range of methods, but also wish to be counseled on the range of available methods. As noted, we found some women sought provider services specifically for one method and did not want to be swayed from their original method choice. As such, family planning counseling services should be balanced to provide a variety of options, while allowing women to ultimately choose their preferred method. Further, our findings noted several women indicated either initial apprehension with family planning services in general or in expressing discordant views with their providers. This finding reinforces the importance of training providers on how to address women’s healthcare needs in contraceptive counseling interactions while valuing women’s preferences surrounding if, when, and what types of contraceptive women wish to use.

## Conclusion

Health providers play a key role in women’s uptake, switching, and discontinuation of family planning methods in Ethiopia. While the majority of interactions were viewed positively, some women reported that their decision to use family planning methods was undermined, as they were directed away from their preferred methods, often toward LARCs. Biased and directive contraceptive counseling limits women’s reproductive autonomy and may affect their satisfaction with family planning services and inhibit their uptake and continued use of family planning methods.

## Supplementary information

**Additional file 1.** Qualitative interview guides and brief quantitative survey.

## Data Availability

The datasets generated and/or analysed during the current study are not publicly available due to the qualitative nature of the data and identifying geographic information, but are available from the corresponding author on reasonable request.

## Abbreviations

AAU: Addis Ababa University
FP2020: Family Planning 2020
FGD: Focus group discussion
IDI: In-depth interview
IRB: Institutional Review Board
JHSPH: Johns Hopkins Bloomberg School of Public Health
LARC: Long-acting reversible contraception
LMIC: Low- and middle-income country
mCPR: Modern contraceptive prevalence rate
PMA2020: Performance Monitoring and Accountability 2020
SDG: Sustainable Development Goal
SRH: Sexual and reproductive health
TFR: Total fertility rate
WGE: Women’s and girls’ empowerment
