# Supplementary material for: “You better use the safer one… leave this one”: the role of health providers in women’s pursuit of their preferred family planning methods
Source: BMC Womens Health. 2020 Aug 12;20:170. doi: 10.1186/s12905-020-01034-1 (PMC7425019; doi:10.1186/s12905-020-01034-1)
Supplement: Supplementary file 1 — Additional file 1. Qualitative interview guides and brief quantitative survey. [file 12905_2020_1034_MOESM1_ESM.pdf]

APPENDIX 1: Female FGD Guide 15-17

PI: Amy Tsui

Study Title: Performance Monitoring and Accountability 2020: Women and Girls' Empowerment Module Qualitative

IRB No: IRB00007909

PI Version Number/Date: v6/June 19, 2017

For facilitator:
Country___________________ Region______________________
Facilitator ID______________ Interview Date________________
Participant IDs____
Is this setting: _____Urban? ______Rural?

Thank you for taking the time to talk with us today. Please remember that everything that we discuss is confidential and should not be shared outside of this group. Let's get started with our discussion. When we talk about community today, we are referring to your close network of friends, family, and neighbors.

1. {autonomy-community} Some families have many children and others have few chidlren. How do people in your community view these differences in family size?
    - *Probe: In what ways might bigger families be viewed differently than smaller families?*
  - Follow-up: What does it mean for women in your community to have a big family? What does it mean for women in your community to have a small family?
    - *Probe: In what ways are they viewed differently?*
  - Follow-up: What about men? What does it mean for men to have a big family, or on a contrary, a small family?
    - *Probe: In what ways are they viewed differently?*
  - Follow-up: Have you ever heard religious leaders in this community talk about how many children a family should have? What do they say?
  - Follow-up: How do people in your community view those who do not have children?
    - *Probe: What about women who are unable to have children?*

2. {autonomy & self-efficacy-couple & individual} In your community, when do women typically have their first child?
  - Follow-up: How is this decision about when a woman has her first child made? Tell me about the process.
    - *Probe: Who participates? How do they discuss this decision?*
    - *Probe: What is the woman's role in the decision?*
    - *Probe: What is the man's role in the decision?*
    - *Probe: What about parents or in-laws?*

- Follow-up: What are some of the reasons women might want to wait to have children?
    - *Probe: Are there any advantages? What about disadvantages?*
    - *Probe: How would people in the community view them?*
- Follow-up: What are some of the reasons men might want to wait to have children?
    - *Probe: Are there any advantages? What about disadvantages?*
    - *Probe: How would people in the community view them?*
- Follow-up: What if a girl gets pregnant and is not married?
    - *Probe: What would happen then?*
    - *Probe: How would she be treated?*
    - *Probe: Partner, parents, friends, people in community.*

3. {autonomy & self-efficacy-couple & individual} What happens if a young woman and her partner don't agree about when to have children? How often does this happen among your friends, family or neighbors?
    - Follow-up: What kinds of things would they disagree about?
        - *Probe: How would they resolve the problem? Who else would get involved?*
    - Follow-up: If a woman didn't have her partner's support, what strategies would she use to avoid pregnancy?
    - Follow-up: How common is it for a young married couple to jointly decide to wait for a few years to have their first child?
    - Follow-up: What pressures or risks would the young married couple face if they waited to have their first child? Who would pressure them?
        - *Probe: Parents, in-laws, other family members, community leaders, peers.*
    - Follow-up: How would they respond to these pressures?

4. {autonomy & self-efficacy-community} Some couples use contraception to control when and how many children they have. How do people in your community view this practice?
        - *Probe for use in relation to delaying, limiting, spacing births.*
        - *Probe: What are the advantages? What are the disadvantages?*
    - Follow-up: What do people say about women who use contraception?
        - *Probe: Unmarried, adolescents, women with no children, women with children.*
    - Follow-up: Would the views of religious leaders or other community leaders here have any influence on a couple's desire to use contraception? How?
        - *Probe: What about parents' views?*
    - Follow-up: Does contraception usually help or hurt the relationship between partners? Why?
    - Follow-up: What methods of family planning do people say good things about? What methods do people say bad things about?
    - Follow-up: With whom do young women usually talk about family planning?
        - *Probe: Can you tell me what these conversations are usually about?*
    - Follow-up: How might these conversations influence young women's thoughts about using or not using family planning?

- Follow-up: How would you describe the type of woman who is able to voice her opinions about contraception?
    - *Probe: Age, education, parity, wealth.*

5. {autonomy & self-efficacy-couple & community} Contraception can relieve couples from the worry of becoming pregnant when they are not prepared for another child. How would members of this community view this benefit?
    - Follow-up: Would it be seen as an important benefit?
        - *Probe: For the man primarily? For the woman primarily? For both?*
    - Follow-up: In this community, would the woman be able to discuss using contraception with her partner?
        - *Probe: How might she talk to him about this? If not, why?*
    - Follow-up: Another benefit of using contraception is that it allows couples to enjoy sex without the worry of pregnancy. Would this beneficial aspect of contraception be more important to the man or to the woman or to both equally? Why?

6. {self-efficacy & agency-individual} What if a young woman in your community decided she wanted to use contraception—how common is that?
        - *Probe: Unmarried, nulliparous women.*
    - Follow-up: What would motivate her to use contraception?
    - Follow-up: Who would be involved in the decision to use contraception?
        - *Probe: Partner, friends, family members.*
    - Follow-up: How would she access contraceptive services? Who would she talk to?
        - *Probe: What could help her access these services?*
    - Follow-up: What might get in the way of her using contraception? What might enable her to use contraception?
        - *Probe: How would the community's views influence her decisions?*
    - Follow-up: What if her partner did not agree with her using contraception? What would happen?
        - *Probe: Why would he not agree? Why would he agree?*
        - *Probe: What about her parents?*
    - Follow-up: What choices does she have if her partner does not agree? What if her parents didn't agree?

7. {autonomy & self-efficacy-community & individual} Now I want to ask about your community's beliefs about sex. How do people view young men who have sex before they marry? What about young women?
        - *Probe: Are there different views for men and women? Why?*
    - Follow-up: How do people view men who have several sexual partners? What about women?
        - *Probe: Are there different views for men and women? Why?*
    - Follow-up: Are there times when men don't want to have sex? What about women?
        - *Probe: Can you give me some examples?*
        - *Probe: How common is this?*
    - Follow-up: From your community's perspective, who can decide when to have sex?

    - *Probe: Man’s decision? Woman’s decision? Joint decision?*
- Follow-up: How does a young man show that he wants to have sex? What about a young woman?
    - *Probe: Is this considered appropriate behavior for the young man? What about the young woman?*
- Follow-up: Can sex be an enjoyable activity for both young men and young women?
    - *Probe: Is it more enjoyable for one over the other?*
- Follow-up: Would it be considered appropriate behavior for the woman to say no to sex to her partner?
    - *Probe: Why or why not?*
- Follow-up: How are women viewed if they show or tell their partners that they want to have sex?

8. {self-efficacy-individual} Sex is part of marriage and relationships. The times when both partners want or do not want to have sex can differ. What options does a woman who wishes to have sex with her partner have when her partner is not interested?
    - Follow-up: What options does she have if her partner wants to have sex and she does not?
    - Follow-up: Have you heard of young women or young men speak about how they can avoid having sex with their partner?
    - Follow-up: What are strategies women use to avoid sex without saying “no”?
    - Follow-up: How might a woman’s partner respond if he realizes that she’s avoiding sex?
        - *Probe: Physical violence, taking away children, isolating from family.*
        - *Probe: Non-violent means, like being supportive/understanding of her decision, attempting to discuss the underlying reasons for her avoidance, negotiating, beg her with gifts.*
    - Follow-up: What would a woman do if she were not able to avoid having sex with her partner? Would she be able to talk to other people about this?
    - Follow-up: Some women feel they must comply with their partner’s desire for sex even if they do not want to have sex. For women who experience this, how can they respond?
        - *Probe: What about with the help of family or friends or others in the community, such as traditional or religious leaders?*

You have been very helpful in sharing your thoughts about reproductive and sexual health and behaviors. Thank you again for your time.

APPENDIX 2: Female FGD Guide 18+

PI: Amy Tsui

Study Title: Performance Monitoring and Accountability 2020: Women and Girls' Empowerment Module Qualitative

IRB No: IRB00007909

PI Version Number/Date: v6/June 19, 2017

For facilitator:
Country___________________ Region______________________
Facilitator ID______________ Interview Date________________
Participant IDs____
Is this setting: _____Urban? ______Rural?

Thank you for taking the time to talk with us today. Please remember that everything that we discuss is confidential and should not be shared outside of this group. Let's get started with our discussion. When we talk about community today, we are referring to your close network of friends, family, and neighbors.

1. {autonomy-community} Some families have many children and others have few children. How do people in your community view these differences in family size?
    - *Probe: In what ways might bigger families be viewed differently than smaller families?*
   - Follow-up: What does it mean for women in your community to have a big family? What does it mean for women in your community to have a small family?
     - *Probe: In what ways are they viewed differently?*
   - Follow-up: What about men? What does it mean for men to have a big family, or on a contrary, a small family?
     - *Probe: In what ways are they viewed differently?*
   - Follow-up: Have you ever heard religious leaders in this community talk about how many children families should have? What do they say?
   - Follow-up: How do people in your community view those who do not have children?
     - *Probe: What about women who are unable to have children?*

2. {autonomy & self-efficacy-couple & individual} In your community, to what extent do couples plan and discuss how many children they want?
    - *Probe: How common is this?*
   - Follow-up: How is the decision made? Tell me about the process.
     - *Probe: Who participates in this decision? How do they discuss this decision?*
     - *Probe: What is the wife's role in the decision?*
     - *Probe: What is the husband's role in the decision?*

- o Follow-up: At what stage of their lives would they typically make this decision?
- o Follow-up: What would be some of the reasons couples would want to plan their family size?
    - o *Probe: Are there any advantages to planning their family size? What about disadvantages?*
- Follow-up: If the woman/wife wants to delay or stop having children, how would she make her husband aware of her intentions?
    - o *Probe: What would she risk by doing this?*

3. {autonomy & self-efficacy-couple & individual} What happens if a husband and his wife do not agree about when to have their next child? How often does this happen among your friends, family or neighbors?
    - Follow-up: How could a woman tell her husband that she doesn't agree with his ideas on when to have their next child? What are her options?
    - Follow-up: What kinds of things would the couple disagree about?
    - Follow-up: How would this be different for young couples versus older couples?
        - o *Probe: Can you explain these differences?*
    - Follow-up: How would they resolve the problem? Who else would get involved?
        - o *Probe: Who would have more power in making the decisions? Why?*
    - Follow-up: If a woman didn't have her husband's support, what strategies would she use to avoid pregnancy?
    - Follow-up: What are the pressures or risks she would face if she didn't get pregnant?
        - o *Probe: Who would pressure her?*
    - Follow-up: How would she respond to these pressures?

4. {autonomy & self-efficacy-community} Some couples use contraception to control when and how many children they have. How do people in your community view this practice?
        - o *Probe for use in relation to delaying, limiting, and/or spacing births.*
        - o *Probe: What are the advantages? What are the disadvantages?*
    - Follow-up: What do people say about women who use contraception?
    - Follow-up: Would the views of religious leaders or other community leaders here have any influence on a couple's desire to use contraception? How?
    - Follow-up: Does contraception usually help or hurt the relationship between a husband and his wife? Why?
    - Follow-up: What methods of family planning do people say good things about? What methods do people say bad things about?
    - Follow-up: With whom do women usually talk about family planning?
        - o *Probe: Can you tell me what these conversations are usually about?*
    - Follow-up: How might these conversations influence people's thoughts about using or not using contraception?
    - Follow-up: How would you describe the type of woman who is able to voice her opinions about contraception?
        - o *Probe: Education, having had a son, parity, wealth.*

5. {autonomy & self-efficacy-couple & community} Contraception can relieve couples from the worry of becoming pregnant when they are not prepared for another child. How would members of this community view this benefit?
   - Follow-up: Would it be seen as an important benefit?
     - *Probe: For the man primarily? For the woman primarily? For both?*
   - Follow-up: In this community, would the wife be able to discuss using contraception with her husband?
     - *Probe: How might she talk to him about this? If not, why?*
   - Follow-up: Another benefit of using contraception is that it allows couples to enjoy sex without the worry of pregnancy. Would this beneficial aspect of contraception be more important to the man/husband or to the woman/wife or to both equally? Why?

6. {self-efficacy & agency-individual} What if a woman in your community decided she wanted to use contraception—how common is that?
     - *Probe: Young women, older women.*
   - Follow-up: What would motivate her to use contraception?
   - Follow-up: Who would be involved in the decision to use contraception?
     - *Probe: Husband, friends, family members.*
   - Follow-up: How would she access contraceptive services? Who would she talk to?
     - *Probe: What could help her access these services?*
   - Follow-up: What might get in the way of her using contraception? What might enable her to use contraception?
     - *Probe: How would the community's views influence her decisions?*
   - Follow-up: What if her husband does not agree with her using contraception?
     - *Probe: Why would he not agree? Why would he agree?*
   - Follow-up: What choices does she have if her husband does not agree?

7. {autonomy & self-efficacy-community & individual} Now I want to ask about your community's beliefs about sex. How do you think this community views men having sex with women inside of marriage? What about outside of marriage?
     - *Probe: Are these different from how the community views married or unmarried women behaving the same way?*
   - Follow-up: How do people view men who have several sexual partners? What about women?
     - *Probe: Are there different views for men and women? Why?*
   - Follow-up: Are there times when men don't want to have sex? What about women?
     - *Probe: Can you give me some examples?*
     - *Probe: How common is this?*
   - Follow-up: From your community's perspective, who can decide when to have sex?
     - *Probe: Man's decision? Woman's decision? Joint decision?*
   - Follow-up: How does a man show that he wants to have sex? What about a woman?
     - *Probe: Is the way a man shows that he wants to have sex considered appropriate behavior for the man? What about the woman?*
   - Follow-up: Can sex be an enjoyable activity for both men and women?

    - *Probe: Is it more enjoyable for one than the other?*
- Follow-up: In what situations is it okay for a man not to want to have sex with his wife?
    - *Probe: What about a woman?*
- Follow-up: How are wives viewed if they show or tell their husbands that they want to have sex?

8. {agency-woman} Sex is part of marriage and relationships. The times when both partners want or do not want to have sex can differ. What options does a woman who wishes to have sex with her husband have when her partner is not interested?
    - Follow-up: What options does she have if her husband/partner wants to have sex and she does not?
    - Follow-up: What happens if wives or husbands disagree on the frequency of having sex?
        - *Probe: How will they resolve the issue?*
    - Follow-up: Would it be considered appropriate behavior for the wife to say no to sex with her husband?
        - Probe: Why or why not?
    - Follow-up: Have you heard wives or husbands talk about how they might avoid having sex with their partner?
    - Follow-up: What are strategies women use to avoid sex without saying "no"?
    - Follow-up: How might a woman's husband respond if he realizes that she's avoiding sex?
        - *Probe: Physical violence, taking away children, isolating from family.*
        - *Probe: Non-violent means, like being supportive/understanding of her decision, attempting to discuss the underlying reasons for her avoidance, negotiating, beg her with gifts.*
    - Follow-up: What would a woman do if she were not able to avoid having sex with her husband? Would she be able to talk to other people about this?
    - Follow-up: Some women feel they must comply with their husband's desire for sex even if they do not want to have sex. For women who experience this, how can they respond?
        - *Probe: What about with the help of family or friends or others in the community, such as traditional or religious leaders?*

You have been very helpful in sharing your thoughts about reproductive and sexual health and behaviors. Thank you again for your time.

APPENDIX 3: Female IDI Guide 15-17

PI: Amy Tsui

Study Title: Women and Girls' Empowerment Module

IRB No: IRB00007909

PI Version Number/Date: v11/July 20, 2017

For interviewer:
Country____________________ Region______________________
Interviewer ID_______________ Participant ID_________________
Interview Date______________
Is this setting: _____Urban? ______Rural?

1. {autonomy-community & individual} Have you ever thought about being pregnant and having children?
    - *Probe: When did you start thinking about this?*
   - Follow-up: What are your thoughts about being pregnant? What are your thoughts on becoming a mother?
     - *Probe: What positive or negative thoughts do you have?*
   - Follow-up: Would having children change the way people treat you? Would it change the way they view you? How?
     - *Probe: Family, friends, people in your community.*
   - Follow-up: What if you didn't have children--would not having children change the way people treat you? Would it change the way they view you? How?
     - *Probe: Family, friends, people in your community.*

2. IF FEMALE HAS A BOYFRIEND/HUSBAND. {autonomy & self-efficacy-couple} Have you had conversations about starting a family with your boyfriend/husband?
   - Follow-up: Can you describe how these conversations occur?
     - *Probe: Who initiates the discussion? What is usually discussed?*
   - Follow-up: Do you feel comfortable having these conversations with your boyfriend/husband? Why or why not?
     - Probe: *What makes you feel comfortable or uncomfortable?*
   - Follow-up: Let's say that you marry and your husband wants a child right away, but you would like to wait. What are your options for waiting?
     - *Probe: How likely is it that you would use one of these options?*
   - Follow-up: Have you and your boyfriend/husband ever talked about <u>not</u> having any children?
     - *Probe: When did you have that conversation? What was it like?*

    - *Probe: Have you talked with him about delaying the first child? Who initiated this conversation? What was it like?*
    - *Probe: Have these conversations with your boyfriend/husband changed since you have been together? In what ways have they changed?*

3. {autonomy-community/family} Who would you talk to about having or not having children? Your family (which relative)? Your friends? Community elders/leaders?
    - *Probe: Reasons for talking to this individual(s)? How would these conversations go?*
   - Follow-up: Do you feel any pressure from these individuals to have or not have children? How do you feel about this?
   - Follow-up: Have you heard of or talked with a religious leader about having or not having children? What was the conversation like?

4. {self-efficacy-individual} Do you feel you can decide when to have your first child?
   - Follow-up: Whose decision do you think it will be when you actually become pregnant? Why?
   - Follow-up: What are your feelings about this decision being yours only or one shared with your partner or family?
   - Follow-up: What about decisions on the timing of future births? Do you think you will make those decisions or will they involve the same people as for the first birth?

5. {autonomy-community & individual} Some women have difficulty getting pregnant right away. Do you ever worry about being able to get pregnant? Please tell me about this.
   - Follow-up: Whom would you talk to about this?
      - *Probe: Role of a husband, family, friends*.
   - Follow-up: Do you think friends, family or a partner would treat or relate with you differently if you were in this situation? How?
      - *Probe: Would their views or behaviors affect you? How?*
   - Follow-up: Do you know if a woman can seek help to enable her to become pregnant? From whom? How common is this?
      - *Probe: If your sister, for example, faced a situation of having difficulty getting pregnant, what advice or options would you share with her?*

6. {autonomy & self-efficacy-community & individual} Some women use contraception or family planning to plan their pregnancies. What do you think about family planning in general?
    - *Probe: What advantages or concerns are there?*
   - Follow-up: Do you think family planning is appropriate for the timing of pregnancies? What about for the spacing of pregnancies? Why or why not?
   - Follow-up: What if you were to become pregnant when you were not ready? What would you do?
      - *Probe: What family planning options would you want to have at that time?*
      - *Probe: What family planning options would you want in the future?*

- Follow-up: Do you know any women in your family, among your friends, or in your community who use contraception?
- Follow-up: Are there specific family planning methods that you know about and like?
  - *Probe: What do you like about them?*
- Follow-up*:* What about methods you know about and dislike*?*
  - *Probe: What do you dislike about them?*
- Follow-up: What do you think about family planning for you?
  - *Probe: Would you ever use it? Why or why not?*
  - *What method would you want to use?*
  - *Probe: Have you ever used it yourself?*

7. {autonomy & self-efficacy-community & couple} Have you had any conversations about family planning with a partner, friends, close relatives or community leaders?
    - *Probe: What were these conversations about?*
    - *Probe: What were their different opinions?*
   - Follow-up: What do your friends generally think about family planning? Do you agree, why or why not?
     - *Probe: What about your relatives? Community or religious leaders?*
   - IF RESPONDENT HAS A BOYFRIEND/HUSBAND. Follow-up: Have you talked to a boyfriend/husband about contraception or family planning?
     - *Probe: What did he say?*
     - *Probe: Why does your boyfriend/husband support or oppose family planning?*
   - IF RESPONDENT HAS A BOYFRIEND/HUSBAND. Follow-up: Have you ever had any disagreement with your boyfriend/husband about family planning?
     - *Probe: What was it about? How did you resolve the disagreement?*

8. SKIP IF RESPONDENT HAS NEVER USED FAMILY PLANNING. {self-efficacy & agency-individual} Let's talk about the first time you decided to use family planning. Can you share with me how you came to that decision?
   - Follow-up: With whom did you discuss this decision?
     - *Probe: Boyfriend/husband, family, friends.*
   - Follow-up: Who was involved in the decision? Who had the final say in the decision?
   - Follow-up: Was anyone opposed to your using family planning? Why were they opposed to it? How did you resolve the disagreement?
   - Follow-up: What method of family planning did you first decide to use? Why did you choose this method?

9. SKIP IF RESPONDENT HAS NEVER USED FAMILY PLANNING OR IS NOT IN A CONSENSUAL UNION. {self-efficacy & agency-individual} Was there a time you used family planning without telling your boyfriend/husband? Can you share how this happened?
   - Follow-up: Why did you decide not to tell him? What were the risks in telling or not telling him?
     - *Probe: Did your boyfriend/husband ever learn about your use? How did he respond?*

10. {autonomy & self-efficacy-individual} Who can best help you decide about using family planning if you are considering it?
    - *Probe: What makes you feel that way?*
- Follow-up: How do you feel about consulting a healthcare provider about family planning? Do you think that healthcare providers have your best interests in health and wellbeing in mind when they counsel you?
- Follow-up: Have you ever been concerned about talking with healthcare providers about family planning? Why?
    - Probe: How would a healthcare provider treat you if you came to ask for family planning?

11. {self-efficacy & agency-couple & individual} Now we'd like to shift and talk about sexual experiences. Sex is an intimate and natural activity in a relationship. If you ever experienced this, can you describe the first time you had sex? If you have not had sex, can you describe how you imagine this will happen the first time?

IF RESPONDENT HAS NEVER HAD SEX:
- Follow-up: Would you know when it was going to happen? How?
- Follow-up: Who would be involved in the decision? Why?
    - *Probe: How would you be involved?*
    - *Probe: What would happen if you said you were not ready?*
    - *Probe: What would happen if you decided you were ready before you got married?*

IF RESPONDENT HAS EVER HAD SEX:
- Follow-up: Did you know when it was going to happen? How?
    - *Probe: (If sexually experienced) Were you told it was going to happen? Did you want it to happen?*
- Follow-up: Did you feel like you had a choice in whether to have sex or not?
    - *Probe: (If responds "no" to follow-up question) Whose decision was it?*
- Follow-up: If you are sexually intimate with your boyfriend/husband now, how do you know when he wants to have sex?
    - *Probe: How does he show or tell you?*
- Follow-up: What about your boyfriend/husband--how does he know if or when you want to have sex?
    - *Probe: How do you show or tell him?*
- Follow-up: What happens if you don't want to have sex?
    - *Probe: How do you communicate this to your boyfriend/husband?*
    - *Probe: How does he react?*
    - *Probe: How often do you not want to have sex? How often does he react this way?*
- Follow-up: Have you ever done something to avoid having sex with your boyfriend/husband? What did you do? How did doing that make you feel?

    - *Probe: How did he respond?*
- Follow-up: Do you feel you have a choice not to have sex if you don't want to?
    - *Probe: What are the consequences if you say no*

You have been very helpful in sharing your thoughts about reproductive and sexual health and behaviors. If you are married, we would like to ask your permission to also interview your husband. The questions that we will ask your husband will be very similar to the ones that we asked you. If you do not want for us to talk to your husband, we will stop and not approach him. Nothing bad will happen to you if you do not want us to interview your husband. We will never tell your husband any of your responses to the questions and all of your information will be kept private. Do we have your permission to approach your husband about an interview?

Thank you for your time.

APPENDIX 4: Female IDI Guide 18+ Nulliparous

PI: Amy Tsui

Study Title: Women and Girls' Empowerment Module

IRB No: IRB00007909

PI Version Number/Date: v6**,** July 20, 2017

For interviewer:
Country____________________ Region______________________
Interviewer ID_______________ Participant ID_________________
Interview Date______________
Is this setting: _____Urban? ______Rural?

1. {autonomy-individual & community} Have you ever thought about pregnancy and having children? When did you start thinking about this? What are your thoughts about being pregnant? About becoming a mother?
    - o *Probe: What are the benefits/advantages of having children? The costs/disadvantages?*
   - Follow-up: How would having children change the way people view you? What about the way people treat you?
     - o *Probe: In your family? What about your husband/partner (if applicable)? What about people in your community?*
   - Follow-up: If you didn't have children, would it change the way people view you? Treat you?
     - o *Probe: In your family? What about your husband/partner (if applicable)? What about people in your community?*

2. IF FEMALE HAS A BOYFRIEND/HUSBAND {self-efficacy-couple & individual} Have you had conversations about starting a family with your husband/partner?
   - Follow-up: Can you describe how these conversations occur?
     - o *Probe: Who initiates?*
     - o *Probe: What is usually discussed?*
     - o *Probe: How often do you have these conversations?*
   - Follow-up: Do you feel comfortable having these conversations with your husband/partner?
     - o Probe: *Why or why not?*
   - Follow-up: Have you both agreed to have children? What happens if either of you disagree about having a child?
     - o *Probe: How are you able to resolve the disagreement?*

- Follow-up: Let's say that your husband/partner wants a child right away, but you would like to wait. What are your options for waiting?
    - *Probe: How likely is it that you would use one of these options?*
- Follow-up: Have you and your husband/partner ever talked about not having any children?
    - *Probe: How long ago did you have that conversation? What was it like?*
- Follow-up: What about a conversation about delaying your first child? What was that conversation like?
- Follow-up: Have these conversations with your husband/partner changed since you have been together? In what ways have they changed?

3. {autonomy-community & family} Who (else) would you talk about having children?
    - *Probe: What were your reasons for talking to this individual (s)?*
    - *Probe: What are these conversations about?*
    - Follow-up: Do you feel any pressure from anyone to have or not have children? Who? How do you feel about this?
    - Follow-up: Have you heard of or talked with a religious leader about having or not having children? What was the conversation like?

4. {self-efficacy-individual} To what extent do you feel you can decide when to have your first child?
    - Follow-up: Whose decision do you think it will be when you actually become pregnant?
        - *Probe: Husband/partner, in-laws/ family, community/religious leaders*
    - Follow-up: What are your feelings about this decision being yours only or one shared with your partner or family?
    - Follow-up: What about decisions on the timing of future births? Do you think those decisions will rest with you or involve the same other persons as the first birth?

5. {autonomy-individual & community} Some women have difficulty getting pregnant right away. Do you ever worry about becoming pregnant? Can you describe a situation when you might have been worried about becoming pregnant?
    - Follow-up: Who did you talk to about this?
        - *Probe: Husband/partner, family, friends.*
    - Follow-up: How did others treat you when you faced this situation?
        - *Probe: How did their views or behaviors affect you?*
    - Follow-up: Can a woman seek help to enable her to become pregnant? From whom? If your sister, for example, faced a situation of having difficulty getting pregnant, what advice or options would you share with her?

6. {autonomy-community & individual/agency-individual} Some women use contraception/family planning to plan their pregnancies. What do you think about family planning in general?
    - *Probe: Any advantages or concerns?*

- Follow-up: Do you think family planning is appropriate for the timing of pregnancies? What about for the spacing of pregnancies? Why or why not?
- Follow-up: What if you were to become pregnant when you were not ready? What would you do?
  - *Probe: What family planning options would you want to have at that time?*
  - *Probe: What family planning options would you want in the future?*
- Follow-up: Are there specific methods you like?
  - *Probe: What do you like about them?*
- Follow-up*:* What about methods you dislike*?*
  - *Probe: What do you dislike about them?*
- Follow-up: What do you think about family planning for you?
  - *Probe: Would you ever use it? Why or why not?*
  - *Probe: What method would you want to use?*
  - *Probe: Have you ever used it yourself?*

7. {autonomy & self-efficacy-community & couple} Have you had any conversations about family planning with friends, close relatives, or community leaders?
    - *Probe: What were these conversations about?*
   - Follow-up: What do your friends generally think about family planning? Do you agree? Why or why not?
     - *Probe: What about your relatives?*
     - *Probe: What about your community or religious leaders?*
   - IF RESPONDENT HAS A BOYFRIEND/HUSBAND. Follow-up: Have you talked to your husband/partner about contraception/family planning?
     - *Probe: What did he say?*
     - *Probe: Why does your husband/partner support / oppose family planning?*
     - *Probe: Have you ever had any disagreement with your husband/partner about family planning? What was it about? How did you resolve the disagreement?*

8. SKIP IF RESPONDENT HAS NEVER THOUGHT ABOUT FAMILY PLANNING FOR HERSELF. {self-efficacy & agency-individual} Let's talk about the first time you decided to use family planning. Can you share with me how you came to that decision?
   - Follow-up: Who did you discuss this decision with?
     - *Probe: Husband/partner, family, friends, community/religious leaders.*
   - Follow-up: Who was involved in the decision? Who had the final say in the decision?
   - Follow-up: Was anyone opposed to you using family planning? Why? How did you resolve the disagreement?
   - Follow-up: What method of family planning did you first decide to use? Why did you choose this method?

9. SKIP IF RESPONDENT HAS NEVER USED FAMILY PLANNING. {self-efficacy & agency-individual} Was there a time you used family planning without telling your husband/partner? Can you share how this happened?

- Follow-up: Why did you decide not to tell him? What were the risks in telling or not telling him?
    - *Probe: Did your husband/partner ever learn about your use?*
    - *Probe: What was his response?*

10. {autonomy & self-efficacy-individual} Who can best help you make a decision about family planning?
    - *Probe: What makes you feel that way?*
- Follow-up: How do you feel about consulting a healthcare provider about family planning?
    - *Probe: Why do you feel this way? Have you ever had a negative/positive experience?*
- Follow-up: Do you think that healthcare providers have your best interest for your health and well-being in mind when they counsel you? think about your best interest?
- Follow-up: Have you ever been concerned when talking with healthcare providers about family planning?

11. {self-efficacy & agency-couple & individual} Now we'd like to shift and talk about sexual experiences. Sex is an intimate and natural activity in a relationship. If you ever experienced this, can you describe the first time you had sex? If you have not had sex, can you describe how you imagine this will happen the first time?

IF RESPONDENT HAS NEVER HAD SEX

- Follow-up: Would you know when it was going to happen? How?
- Follow-up: Who would be involved in the decision? Why?
    - *Probe: How would you be involved?*
    - *Probe: What would happen if you said you were not ready?*
    - *Probe: What would happen if you decided you were ready before you got married?*

IF RESPONDENT HAS EVER HAD SEX

- Follow-up: Did you know it was going to happen? How?
    - *Probe: Were you told it was going to happen?*
    - *Probe: Did you want it to happen?*
- Follow-up: Did you feel like you had a choice in whether to have sex or not?
    - Probe*: If responds "no"*: Whose decision was it?
- Follow-up: When you have sex with your husband/partner now, how do you know when he wants to have sex?
    - *Probe: Does he show or tell you?*
- Follow-up question: What about your husband/partner--how does he know you want to have sex?
    - *Probe: How do you show or tell him?*
- Follow-up: What happens if you don't want to have sex?
    - *Probe: How do you communicate this to your husband/partner?*

  - *Probe: How does your husband/partner react?*
  - *Probe: How often do you not want to have sex? How often does he react this way?*
- Follow-up*:* Have you ever done something to avoid having sex with your husband/partner?
  - *Probe: How did your husband/partner respond?*
- Follow-up: Do you feel you have a choice not to have sex if you don't want to?
  - *Probe: What happens if you say "no"?*

You have been very helpful in sharing your thoughts about reproductive and sexual health and behaviors. If you are married, we would like to ask your permission to also interview your husband. The questions that we will ask your husband will be very similar to the ones that we asked you. If you do not want for us to talk to your husband, we will stop and not approach him. Nothing bad will happen to you if you do not want us to interview your husband. We will never tell your husband any of your responses to the questions and all of your information will be kept private. Do we have your permission to approach your husband about an interview?

Thank you again for your time.

APPENDIX 5: Female IDI Guide 18+ Parous

PI: Amy Tsui

Study Title: Women and Girls' Empowerment Module

IRB No: IRB00007909

PI Version Number/Date: v7/July 20, 2017

For interviewer:
Country____________________ Region______________________
Interviewer ID_______________ Participant ID_________________
Interview Date______________
Is this setting: _____Urban? ______Rural?

1. {autonomy/self-efficacy-individual} Let's think back to when you were a young girl and had not yet started a family. When did you start thinking about having children? What did you think motherhood would be like?
   - Follow-up: Who did you talk to about these issues? What were these conversations about?
     - *Probe: Family, in-laws, friends, community/religious leaders.*
   - Follow-up: To what extent could you decide when to have your first child? How much of the timing was your decision? Whose decision was it largely?
     - *Probe: What were the reasons? How did you feel about this?*

2. {autonomy-community & couple} Now think back to the first time you became pregnant. Did being pregnant change the way people treated you? How?
     - *Probe: Husband/partner, family, in-laws, friends*
   - Follow-up: How did becoming a mother change your role/position in the family?
     - *Probe: Can you give me an example?*
   - Follow-up: After becoming a mother, did you have more or less say in family decisions?
     - *Probe: What kinds of family decisions?*
     - *Probe: How did you feel about being more/less included in family decisions?*
   - Follow-up: Would you say becoming a mother changed how people valued you?
     - *Probe: In what ways?*
     - *Probe: Husband/partner, family, in-laws, friends.*
   - Follow-up: Would you say people treated you differently because you had a child?
     - *Probe: How so?*
   - Follow up: How did your relationship with your husband/partner change after you became a mother?
     - *Probe: Can you give me an example?*

3. {autonomy-individual} Now I want to ask about your own thoughts about becoming a mother. How did you feel about becoming a mother the first time?
   - Follow-up: How did becoming a mother change the ways you thought about yourself?
     - o *Probe: In what ways?*
     - o *Probe: Would you say you valued yourself differently? How so? Can you give an example?*

4. {self-efficacy-individual} After having your first child, what were your thoughts about having more children?
   - Follow-up: How soon after your first child did you start to think about the next pregnancy/child? Did you feel you had more/less say over the timing of the next pregnancy/birth?
   - Follow-up: How did your thoughts on having more children change over time with either more pregnancies or more time passing since the last birth? In what ways did they change?
     - o *Probe: Number of children, changes in relationship, age.*
     - o *Probe: Were there any pregnancies that were particularly difficult? Did this impact your feelings on having more children?*
   - Follow-up: Did you experience any pressure to get pregnant again? From whom?
     - o *Probe: Husband/partner, in-laws/other relatives, friends, religious leaders.*
   - Follow-up: How did you feel about that pressure? How did you manage that pressure?
     - o *Probe: Can you give me an example?*

5. {self-efficacy-couple & individual} After you had your first child, did you have conversations with your husband/partner about having more children?
     - o *Probe: How often did you have these conversations?*
     - o *Probe: What were these conversations about?*
   - Follow-up: Did you feel comfortable having these conversations with your husband/partner?
     - o *Probe: Why or why not?*
   - Follow-up: Did you both agree on having more children? Were there any times you did not agree with your husband/partner? Did the disagreement ever end up in arguments?
     - o *Probe: What was the disagreement about?*
     - o *Probe: How were you able to resolve the disagreement?*
   - Follow-up: Did you and your husband/partner ever talk about <u>not</u> having more children?
     - o *Probe: When did you have that conversation?*
     - o *Probe: What was the conversation like?*
     - o *Probe: What about a conversation about delaying a pregnancy? What was that conversation like?*
   - Follow-up: Did these conversations with your husband/partner change over time? In what ways did they change?
     - o *Probe: Number of children, length of relationship, age.*
   - Follow-up: If you do not want to have a child right away, but your husband/partner does, what options do you have to delay a pregnancy?
     - o *Probe: Did this ever happen? Can you describe the situation?*

- *Probe: How would or did you handle this with him?*
- *Probe: Did/Would you talk to him about it? Did/Would you talk to anyone else?*

6. {autonomy-community & family} Did you involve other family or community members in your decisions about number of children?
    - *Probe: Can you give me an example?*
    - *Probe: How did you feel about these discussions?*
- Follow-up: Did your involvement of others in decisions about how many children you would have change over time, that is, more or less involvement? At what point did their role change?
    - *Probe: Number of children, length of marriage, age, other circumstances*

7. {autonomy-community & individual} Some women have difficulty getting pregnant right away. Did you ever worry about becoming pregnant? Can you describe that situation?
- Follow-up: Who did you talk to about this?
    - *Probe: Husband/partner, family, friends, community/religious leaders*.
- Follow-up: How did these others treat you or relate with you when you faced this situation?
    - *Probe: How did their views or behaviors affect you?*
- Follow-up: Did you seek out any help to enable you to become pregnant? From whom? If this situation happened to a sister, for example, what advice or options would you share with her?

8. {autonomy-individual} Some women use contraception/family planning to plan their pregnancies. What do you think about family planning in general?
    - *Probe: Any advantages or concerns?*
- Follow-up: Do you think family planning is appropriate for the timing of pregnancies? What about for the spacing of pregnancies? Why or why not?
- Follow-up: What if you were to become pregnant when you were not ready? What would you do?
    - *Probe: What family planning options would you want to have at that time?*
    - *Probe: What family planning options would you want in the future?*
- What family planning options would you have for the future or present?
- Follow-up: Are there specific family planning methods you like?
    - *Probe: What do you like about them?*
- Follow-up*:* What about methods you dislike*?*
    - *Probe: What do you dislike about them?*
- Follow-up question: What do you think about family planning for you?
    - *Probe: Would you ever use it? Why or why not?*
    - *What method would you want to use?*
    - *Probe: Have you ever used it yourself?*

9. {autonomy & self-efficacy-community & couple} Have you had any conversations about family planning with friends, close relatives, or community leaders?
    - *Probe: What were these conversations about?*

  - *Probe: How often have these conversations occurred?*
  - *Probe: Is family planning easy to talk about?*
- Follow-up: What do your friends generally think about family planning? Do you agree with your friends' views? Why or why not?
  - *Probe: What about your relatives? Do you agree with your relatives' views? Why or why not?*
  - *Probe: What about community or religious leaders? Do you agree with their views? Why or why not?*
- Follow-up: Have you talked to your husband/partner about contraception/family planning?
  - *Probe: What did he say? How did the conversation begin?*
  - *Probe: Why does your husband/partner support / oppose family planning?*
  - *Probe: Have you ever had any disagreement with your husband/partner about family planning? What was the disagreement about? How did you resolve the disagreement?*

10. SKIP IF RESPONDENT HAS NEVER THOUGHT ABOUT FAMILY PLANNING FOR HERSELF {self-efficacy & agency-individual} Let's talk about the first time you decided to use family planning. Can you share with me how you made that decision?
- Follow-up: Who did you discuss this decision with?
  - *Probe: Husband/partner, family, friends.*
- Follow-up: Who was involved in the decision? Who had the final say?
- Follow-up: Was anyone opposed to you using family planning? Why? How did you resolve the disagreement?
- Follow-up: What method of family planning did you first decide to use? Why did you choose this method?

11. SKIP IF RESPONDENT HAS NEVER USED FAMILY PLANNING. {self-efficacy & agency-individual} Was there a time you used family planning without telling your husband/partner? Can you share how this happened?
- Follow-up: Why did you decide not to tell him? What were the risks in telling or not telling him?
  - *Probe: Did your husband/partner ever learn about your use?*
  - Probe*: What was his response?*

12. {autonomy & self-efficacy-individual} Who can best help you make a decision about family planning if you are considering its use?
  - *Probe: What makes you feel that way?*
- Follow-up: How do you feel about consulting a healthcare provider about family planning?
  - *Probe: Why do you feel this way? Have you ever had a negative/positive experience?*
- Follow-up: Do you think that healthcare providers have your best interest for your health and well-being in mind when they counsel you?
- Follow-up: Have you ever been concerned about talking with healthcare providers about family planning? Why?

13. {self-efficacy & agency-couple & individual} Now, I would like to shift and talk about when you and your husband/partner have sex. Sex is an intimate and natural activity in a marriage/relationship. Can you describe your first sexual experience?
    - Follow-up: Did you know it was going to happen? How?
        - *Probe: Were you told it was going to happen?*
        - *Probe: At that time, did you want it to happen?*
    - Follow-up: Did you feel like you had a choice in whether to have sex or not?
        - *Probe: If responds "no": Whose decision was it?*
    - Follow-up: When you have sex with your husband/partner now, how do you know when he wants to have sex?
        - *Probe: How does he show or tell you?*
    - Follow-up: What about your husband/partner--how does he know you want to have sex?
        - *Probe: How do you show or tell him?*
    - Follow-up: What happens if you don't want to have sex?
        - *Probe: How do you communicate this to your husband/partner?*
        - *Probe: How does your husband/partner react?*
        - *Probe: How often do you not want to have sex? How often does he react this way?*
    - Follow-up*:* Have you ever done something to avoid having sex with your husband/partner?
        - *Probe: How did your husband/partner respond?*
    - Follow-up: Do you feel you have a choice not to have sex if you don't want to?
        - *Probe: What happens if you say 'no'?*

You have been very helpful in sharing your thoughts about reproductive and sexual health and behaviors. If you are married, we would like to ask your permission to also interview your husband. The questions that we will ask your husband will be very similar to the ones that we asked you. If you do not want for us to talk to your husband, we will stop and not approach him. Nothing bad will happen to you if you do not want us to interview your husband. We will never tell your husband any of your responses to the questions and all of your information will be kept private. Do we have your permission to approach your husband about an interview?

Thank you again for your time.

APPENDIX 6: Male FGD Guide

PI: Amy Tsui

Study Title: Performance Monitoring and Accountability 2020: Women and Girls' Empowerment Module Qualitative

IRB No: IRB00007909

PI Version Number/Date: v1/June 3, 2017

For facilitator:
Country___________________ Region______________________
Facilitator ID______________ Interview Date________________
Participant IDs____
Is this setting: _____Urban? ______Rural?

Thank you for taking the time to talk with us today. Please remember that everything that we discuss is confidential and should not be shared outside of this group. Let's get started with our discussion. When we talk about community today, we are referring to your close network of friends, family, and neighbors.

1. {autonomy-community} Some families have many children and others have few children. How do people in your community view these differences in family size?
    - o *Probe: In what ways might bigger families be viewed differently than smaller families?*
    - Follow-up: What does it mean for men in your community to have a big family? What does it mean for men in your community to have a small family?
        - o *Probe: In what ways are they viewed differently?*
    - Follow-up: What about women? What does it mean for women to have a big family? What about a small family?
        - o *Probe: In what ways are they viewed differently?*
    - Follow-up: How do people in your community view those who do not have children?
        - o *Probe: What about women who are unable to have children?*
    - Follow-up: Have you ever heard religious leaders in this community talk about how many children a family should have? What do they say?

2. {self-efficacy-couple} In your community, to what extent do couples plan and discuss how many children they want?
    - o *Probe: How common is this?*
    - o *Probe: Tell me about the process. Who participates in this decision? How do they discuss this decision?*
    - Follow-up: Which partner has more say in deciding when to have the next child? Why? How does that person show his or her influence in this decision?

- Follow-up: At what stage of their lives would the couple typically make this decision?
- Follow-up: What would be some of the reasons couples would want to plan their family size?
    - *Probe: Are there any advantages to planning their family size? What about disadvantages?*
- Follow-up: How do husbands know that their wife wants to delay or stop having children?
    - *Probe: Does she show or tell him?*

3. {autonomy & self-efficacy-couple & individual} What happens if a husband and his wife do not agree about when to have their next child? How often does this happen among your friends, family or neighbors?
    - Follow-up: What kinds of things would the couple disagree about?
    - Follow-up: How would this be different for young couples versus older couples?
        - *Probe: Can you explain these differences?*
    - Follow-up: How would they resolve the problem? Who else would get involved?

4. {autonomy-community} Some couples use contraception to control when and how many children they have. How do people in your community view couples that use contraception?
        - *Probe for use in relation to delaying, limiting, and/or spacing births.*
        - *Probe: Does this differ for men and women?*
    - Follow-up: What do people say about women who use contraception?
    - Follow-up: Would the views of religious leaders or other community leaders here have any influence on a couple's desire to use contraception? How?
    - Follow-up: Does contraception usually help or hurt the relationship between the husband and wife? Why?
    - Follow-up: What methods of family planning do people say good things about? What methods do people say bad things about?
    - Follow-up: Do men ever talk about family planning with their peers? What do they talk about?

5. {autonomy & self-efficacy-couple & community} Contraception can relieve couples from the worry of becoming pregnant when they are not prepared for another child. How would members of this community view this benefit?
    - Follow-up: Would it be seen as an important benefit?
        - *Probe: For the man primarily? For the woman primarily? For both?*
    - Follow-up: In this community, would the wife be able to discuss using contraception with her husband?
        - *Probe: How might she talk to him about this? If not, why?*
    - Follow-up: Another benefit of using contraception is that it allows couples to enjoy sex without the worry of pregnancy. Would this beneficial aspect of contraception be more important to the man/husband or to the woman/wife or to both equally? Why?

6. {self-efficacy & agency-individual} What if a woman in your community decided she wanted to use contraception? How common is that?
        - *Probe: Young women, older women.*
    - Follow-up: What would motivate her to use contraception?
    - Follow-up: Who would be involved in the decision to use contraception?
    - Follow-up: What might get in the way of her using contraception? What might enable her to use contraception?
        - *Probe: How would the community's views influence her decisions?*
    - Follow-up: How would men help or hurt women's access to family planning services?
    - Follow-up: How would you describe the type of woman who is able to voice her opinions about contraception?
        - *Probe: Education, having had a son, parity, wealth.*
    - Follow-up: What about women who decide to use contraception without telling their husbands?
        - *Probe: How common is this? When does this happen?*
    - Follow-up: When would this be considered appropriate behavior?

7. {autonomy & self-efficacy-community & individual} Now I would like to talk with you about the beliefs of members in this community about sexual behaviors. How do you think this community views men having sex with women inside of marriage? What about outside of marriage?
    - Follow-up: Are these different from how the community views married or unmarried women behaving the same way?
    - Follow-up: How do people view men who have several sexual partners? What about women?
        - *Probe: Are there different views for men and women? Why?*
    - Follow-up: From your community's perspective, who can decide when to have sex?
        - *Probe: Is it a man's decision? Woman's decision? Joint decision?*
    - Follow-up: How does a man show that he wants to have sex? What about women?
        - *Probe: Is this way of showing that he wants to have sex considered appropriate behavior for the man? What about the woman?*
    - Follow-up: Can sex be an enjoyable activity for both men and women? Is it more enjoyable for one over the other?
    - Follow-up: In what situations is it okay for a man not to want to have sex with his wife?
        - *Probe: What about a woman?*
    - Follow-up: What would be acceptable reasons for not wanting to have sex for the man /husband? What about for the woman/wife?
    - Follow-up: What happens if wives or husbands disagree on how often they have sex?
        - *Probe: How will they resolve the issue?*
    - Follow-up: Have you heard wives or husbands talk about how they might avoid having sex with their partner?
        - *Probe: What do women say? What do men say?*

- Follow-up: How would a man by viewed in your community if others knew he needed to negotiate with his wife in order to have sex?
    - *Probe: What would his friends say, what about his family?*
    - *How would he respond to this situation?*
- Follow-up: How might a woman's husband respond if he realizes that she's avoiding sex?
    - *Probe: Physical violence, taking away children, isolating from family.*
    - *Probe: Non-violent means, like being supportive/understanding of her decision, attempting to discuss the underlying reasons for her avoidance, negotiating, beg her with gifts.*

You have been very helpful in sharing your thoughts about reproductive and sexual health and behaviors. Thank you again for your time.

APPENDIX 7: Male IDI Guide

PI: Amy Tsui

Study Title: Performance Monitoring and Accountability 2020: Women and Girls' Empowerment Module Qualitative

IRB No: IRB00007909

PI Version Number/Date: v5/June 19, 2017

For interviewer:
Country___________________ Region_____________________
Interviewer ID______________ Participant ID________________
Interview Date_____________
Is this setting: _____Urban? ______Rural?

NOTE FOR INTERVIEWER: If the respondent has multiple wives, determine which wife the respondent will be talking about today. Record her name and use throughout.
Wife's Name___________________

1. {autonomy & self-efficacy-individual & couple} Let's think back to when you were a young boy and had not yet started a family. When did you start thinking of having children? What did you think fatherhood would be like?
   - Follow-up: Who did you talk to about these issues? What were these conversations about?
     - *Probe family, in-laws, friends, community/religious leaders.*
   - Follow-up: To what extent, do you feel you had any control over when to have your first child?
     - *Probe: Whose decision was it? Why?*
     - *Probe: How did you feel about this?*
   - Follow-up: Did you feel your wife had any control over when you had your first child?
     - *Probe: How did that make you feel?*

2. {autonomy-community} The first time your first wife became pregnant, how did having a child change the way people treated you?
     - *Probe: Wife, family, in-laws, friends, community/religious leaders.*
   - Follow-up: How did becoming a father change your role in the family?
     - *Probe: Can you give me an example?*
   - Follow-up: How did it change your role with your wife?
     - *Probe: Can you give me an example?*
   - Follow-up: Would you say becoming a father changed how people valued you?
     - *Probe: In what ways?*
     - *Probe: Wife, family, in-laws, friends, community/religious leaders.*

- *Probe: Did it change the way your wife valued you?*

3. {autonomy–community & couple} Did becoming pregnant change the way people treated or valued your wife?
    - *Probe: Family, in-laws, friends, community/religious leaders.*
    - *Probe: Can you give an example?*
   - Follow-up: Did it change the way you treated or valued her?
     - *Probe: Can you give an example of how you treated her differently?*
   - Follow-up: Did it change the way she interacted with you?
     - *Probe: In what ways?*
   - Follow-up: Did her getting pregnant change your relationship?
     - *Probe: How did your relationship change?*
   - Follow-up: Would you say having a child changed your wife's role in the family?
     - *Probe: In what ways did her role change?*
     - *Probe: Can you give me an example?*

4. IF ONLY 1 WIFE. {autonomy & self-efficacy-couple} After having your first child, what were your thoughts about having more children?
   - Follow-up: How did these thoughts change over time? In what ways did they change?
     - *Probe: What made them change?*
     - *Probe: Number of children, age, length of relationship.*
   - Follow-up: Did you feel pressured to have more children?
     - *Probe: Who pressured you? Why?*
     - *Probe: Wife, in-laws, family*
   - Follow-up: Did you ever pressure your wife to have more children?
     - *Probe: Why?*
     - *Probe: What was her response?*
     - *Probe: How did you manage the situation?*

5. IF MULTIPLE WIVES. {autonomy & self-efficacy-couple} After having your first child, what were your thoughts about having more children?
   - Follow-up: How did these thoughts change over time? In what ways did they change?
     - *Probe: What made them change?*
     - *Probe: Number of children, number of wives, age, length of relationship.*
   - Follow-up: Did you ever discuss with your first wife how many children you wanted together?
     - *Probe: Did you agree or disagree about this issue?*
   - Follow-up: Did marrying again change your plans about having children?
     - *Probe: With your first wife?*
     - *Probe: With your new wife?*
   - Follow-up: Have you ever felt pressured to have additional children? Who pressured you?
     - *Probe: Wives, in-laws, family, community/religious leaders.*
   - Follow-up: Did you ever pressure one of your wives to have additional children?
     - *Probe: Why?*
     - *Probe: What was her response?*
     - *Probe: How did you manage the situation?*

6. {self-efficacy-couple & individual} After you had your first child, did you have conversations with your wife/wives about having more children?
    - o *Probe: What were these conversations about?*
   - Follow-up: Are there any times you disagreed about this issue?
     - o *Probe: What was the disagreement about?*
     - o *Probe: How were you able to resolve the disagreement?*
   - Follow-up: Did you and your wife ever talk about delaying or stopping having children?
     - o *Probe: Why did you want to delay or stop having children?*
     - o *Probe: Why did your wife want to delay or stop having children?*
   - Follow-up: Did these conversations change over time? In what ways did they change?
     - o *Probe: Number of children, number of wives, age*

7. {autonomy-community & family} How did you involve other family members into these decisions?
    - o *Probe: Can you give me an example?*
    - o *Probe: How did you feel about these discussions?*
   - Follow-up: Did your role in these decisions ever change? At what point did your role change? What about your wife?
     - o *Probe: Number of children, length of marriage, age, other circumstances*

8. {autonomy-community & individual} Some couples have difficulty having children. Can you describe a situation when you might have been worried about this?
   - Follow-up: Who did you talk to about this?
     - o *Probe: Wife, family, friends*, community leaders
   - Follow-up: How did this affect your relationship with your wife?
   - Follow-up: How did others treat you when you faced this situation?
     - o *Probe: How did this view affect you?*
   - Follow-up: How did your wife treat you when you faced this situation?
     - o *Probe: How did this view affect you?*

9. {agency-couple} Some men use contraception/family planning with their wives/ partners to plan when they have children. What do you think about family planning in general?
    - o *Probe: Are there any advantages or concerns with using family planning?*
    - o *Probe: Are there advantages or disadvantages in using family planning for women? For men?*
   - Follow-up: What do you think about family planning for you and your wife?
     - o *Probe: Why are you supportive/opposed to it?*

10. {autonomy & self-efficacy-community & couple} Have you had any conversations about family planning with friends, close relatives, or community/religious leaders?
    - o *Probe: What were these conversations about?*
    - o *Probe: Are there any methods you like/dislike?*
   - Follow-up: What do your friends generally think about family planning?
     - o *Probe: Do you agree with your friends' views? Why or why not?*

        - *Probe: What about your family/in-laws? Do you agree with their views? Why or why not?*
        - *Probe: What about your community/religious leaders? Do you agree with their views? Why or why not?*
    - Follow-up: Have you talked to your wife/wives about family planning?
        - *Probe: What were these conversations about?*
        - *Probe: Have you ever had any disagreement about family planning? What was the disagreement about?*
        - *Probe: How did you resolve the disagreement?*

11. {self-efficacy-community & couple} Have you had any experience with family planning?
        - *Probe: What were these experiences like?*
    - Follow-up: The first time you used family planning with your wife, who was involved in the decision?
        - *Probe: wife, family, friends, community leaders.*
    - Follow-up: What was your point of view on using family planning? What was your wife's point of view?
    - Follow-up: Who had the final word? Why?
    - Follow-up: Was anyone opposed to you using family planning? Why?
    - Follow-up: What method of family planning did you first decide to use?
        - *Probe: Why did you choose this method?*

12. SKIP IF RESPONDENT HAS NEVER USED FAMILY PLANNING. {self-efficacy & agency-individual} Was there a time your wife used family planning without telling you first? Can you explain how that happened?
    - Follow-up: Why did she decide not to tell you?
    - Follow-up: How did you find out?
    - Follow-up: What was your reaction?

13. {self-efficacy & agency-couple & individual} Now, I would like to shift and talk about when you and your wife have sex. Sex is an intimate and natural activity in a marriage/relationship. Can you describe your first sexual experience?
    - Follow-up: Did you know it was going to happen? How?
        - *Probe: At the time, did you want it to happen?*
        - *Probe: At the time, did your wife/partner want it to happen?*
    - Follow-up: Did you feel like you had a choice in whether to have sex or not?
        - *Probe: If responds "no": Whose decision was it?*
        - *Probe: Did you feel like your wife/partner had a choice in whether to have sex or not?*
    - Follow-up: When you have sex with your wife now, how does she know you want to have sex?
        - *Probe: How do you show or tell her?*
    - Follow-up: What about you--how do you know your wife wants to have sex?
        - *Probe: How does she show or tell you?*

- Follow-up: What happens if you don’t want to have sex?
    - *Probe: How often does this happen?*
    - *Probe: How does your wife react?*
- Follow-up: What happens if your wife doesn’t want to have sex?
    - *Probe: How often does this happen?*
    - *Probe: How do you react?*
- Follow-up: Do you feel you have a choice not to have sex if you don’t want to? Do you feel your wife has a choice not to have sex if she doesn’t want to?
    - *Probe: Why or why not?*

You have been very helpful in sharing your thoughts about reproductive and sexual health and behaviors. Thank you again for your time.

APPENDIX 8: Brief Survey For In-Depth Interview And Focus Group Discussion

---

PI: Amy Tsui

Study Title: Women and Girls' Empowerment Module

IRB No: IRB00007909

PI Version Number/Date: v6/June 19, 2017

For interviewer:

Country____________________ Region______________________

Interviewer ID_______________ Participant ID_________________

Interview Date______________ Village:______________________

Is this setting: _____Urban? ______Rural?

For participant:

1. What is your sex?
   - Male 0
   - Female 1
2. How old are you? ____________ *years*
3. Are you currently married or living together with a man/woman as if married?
   - No, never in union 0
   - Yes, currently married 1
   - Yes, living with a man 2
   - Not currently in union: Divorced / separated 3
   - Not currently in union: Widow 4
4. How long have you been living with your wife/husband or partner?
   - *Month*__________
   - *Year*___________
5. What is the highest level of school you attended?
   - Never attended 0
   - Primary 1
   - Secondary 2
   - Higher 3
6. How many times have you/your wife given birth? ______ *number of times*
7. Does your husband / partner have other wives or does he live with other women as if married? If male, do you have other wives or live with other women as if married?
   - Yes 1
   - No 0
   - Don't know ....-88
8. What language do you speak at home? ________________
